# Pamphlet Notices

**Published:** 1870-12

**Authors:** 


### Pamphlets.

*Portraits of Diseases of the Skin.*

Messrs. Lindsay & Blakiston send us Part I of A Descriptive Catalogue of the New Sydenham Society's Atlas of Portraits of Diseases of the Skin. Compiled at the request of the Council, by Jonathan Hutchinson, F. R. C. S., etc., etc. They announce that they are now prepared to receive subscriptions for the publications of the New Sydenham Society for the year 1870, at ten dollars, payable in currency, and invariably in advance, and to furnish any of the previous years at the same rate and on the same terms. The practical character and permanent value of these publications, and the very low price at which they are furnished, commend them to the favorable attention of the medical profession in the United States.

Forty-four volumes were published during the eleven years up to, and including, 1869.

The works to be published in 1870 are Trousseau's Clinical Medicine, vol. iii; Stricker's Manual of Histology, vol. i; Niemeyer on Phthisis; Wunderlich's Treatise on the Use of the Thermometer in Disease; and A Tenth Fasciculus of the Atlas of Skin Diseases.

Subscribers at a distance can have their volumes mailed to them, postage paid, as they appear, by remitting $1.50 in addition to the subscription price for the year.

Non-subscribers can obtain the books published during any one year by subscribing and paying for that year $10, but no volumes or books can be had otherwise or separately except the following: The Year Books for 1859, '60, '61, and 62, for $10.50; Portraits of Skin Diseases, Fasciculi 1 to 9, for $42. A Descriptive Catalogue of the Society's Atlas of Portraits of Diseases of the Skin, and their last Report, will be furnished gratis upon application.

From the same Publishers, by Cobb Brothers, 81 and 83 Lake Street, we receive the

*Physician's Visiting List for* 1870.

This visiting list is the simplest, and by many physicians preferred to any other form published. It is too well known, having now been before the profession for twenty years, to require description. The extended circulation it has achieved enables the publishers the present year to make a material reduction in its price. For convenience of our readers we give the sizes and prices at which copies can be obtained by addressing the publishers, or any of the principal booksellers. Mailed post-paid on receipt of price.

For 25 Patients weekly, tucks, pockets and pencil, $1; for 50 Patients weekly, tucks, pockets and pencil, $1.25; for 75 Patients weekly, tucks, pockets and pencil, $1.50; for 100 Patients weekly, tucks, pockets and pencil, $2; for 50 Patients weekly, 2 vols. Jan. to June, July to Dec., tucks, pockets and pencil, $2.50; for 100 Patients weekly, 2 vols. Jan. to June, July to Dec., tucks, pockets and pencil, $3; for 25 Patients weekly, interleaved, tucks, pockets and pencil, $1.50; for 50 Patients weekly, interleaved, tucks, pockets and pencil, $1.75; for 50 Patients weekly, interleaved, 2 vols. Jan. to June, July to Dec., tucks, pockets and pencil, $3.

*Photographic Review of Medicine and Surgery.* A Bi-Monthly Illustration of Interesting Cases, accompanied by Notes; edited by F. F. MAURY, M.D., and L. A. DUHRING, M.D. No. 1, Vol. 1, October, 1870. Contents: Multilocular Hydatid Tumor, S. D. Gross, M.D.; Meningocele, D. Hayes Agnew, M.D.; Horny Tumors, Wm. H. Pancoast, M.D.; Keloid Tumor, F. F. Maury, M.D. Published by J. B. Lippincott & Co., 715 and 717 Market Street, Philadelphia. $6 per annum, in advance.

From the Prospectus we extract:

The Photographic Review will appear in numbers, every other month, each containing four Photographic Plates, with appropriate notes and remarks. At the end of the year the six numbers may be bound together, forming a handsome volume.

We take pleasure in announcing that Drs. S. D. Gross, Joseph Pancoast, D. Hayes Agnew, R. J. Levis, A. Hewson, Wm. Hunt, J. H. Brinton, John H. Packard, Wm. Pepper, J. M. Da Costa, T. G. Morton, Wm. H. Pancoast, S. Weir Mitchell, S. W. Gross, Harrison Allen, J. Ashhurst, Jr., and other eminent members of the profession, are among the regular contributors.

The initial No. which is before us fully sustains the promise of the publishers. The enormous expense necessaiily incurred in the production of the very elegant and striking photographs which accompany the descriptive text, must demand a large subscription list for its support. We sincerely trust the undertaking may meet with the success it eminently deserves. It is creditable in every way to the enterprising originators, and to the profession. The photographs alone are worth a dollar each.

*Constitution and By-Laws of the Medical Library and Journal Association of New York, with List of Officers and Members.* 1870.

